# 

**Published:** 1892-06

**Authors:** 


### Communications.

TO THE DENTAL PROFESSION.—For more than a year I have been using in my practice in this city a method for obtunding sensitiveness in dentine, which is original with myself and wholly unlike any process used hitherto for this purpose. I have now so far perfected my appliance as to be able to announce in the face of your incredulity, that *I have found a way to avoid every vestige of pain in filling teeth*, with complete safety to the pulp and to the absolute satisfaction of the patient. All dentists will readily appreciate the effect of this unique auxiliary upon my practice. Through its instrumentality my patronage has increased until now eight dental chairs are used, and I have established its value beyond question, not alone to my patients in the relief from suffering, but to myself as a money winner. I am lead, therefore, to offer my new method for painless filling to the profession, and one dentist in each town or city can secure the same by purchase. Applications will be favorably noticed in the order in which they are received. Address all communications to

C. E. HALE, D. D. S.,

St. Paul, Minn.

